# Supplementary material for: A Dataset for Evaluating Contextualized Representation of Biomedical Concepts in Language Models
Source: Sci Data. 2024 May 4;11:455. doi: 10.1038/s41597-024-03317-w (PMC11069517; doi:10.1038/s41597-024-03317-w)
Supplement: Supplementary file 1 — Supplementary information [file 41597_2024_3317_MOESM1_ESM.docx]

Evaluating two pre-processing strategies on the BioWiC test set

Supplementary material

| Model | Term identity | | Abbereviations | | Synonyms | | Label similarity | | All | |
| --- | --- | --- | --- | --- | --- | --- | --- | --- | --- | --- |
|  | ‘‘ ’’ | « » | ‘‘ ’’ | « » | ‘‘ ’’ | « » | ‘‘ ’’ | « » | ‘‘ ’’ | « » |
| BERT | 0.63 | 0.70 | 0.82 | 0.70 | 0.72 | 0.69 | 0.83 | 0.86 | 0.70 | 0.71 |
| ELECTRA | 0.64 | 0.65 | 0.70 | 0.72 | 0.68 | 0.68 | 0.82 | 0.80 | 0.68 | 0.68 |
| RoBERTa | 0.67 | 0.67 | 0.77 | 0.78 | 0.72 | 0.73 | 0.82 | 0.86 | 0.71 | 0.72 |
| BioBERT | 0.71 | 0.67 | 0.86 | 0.88 | 0.76 | 0.78 | 0.84 | 0.86 | 0.76 | 0.75 |
| Bio_ClinicalBERT | 0.61 | 0.64 | 0.84 | 0.82 | 0.73 | 0.72 | 0.84 | 0.85 | 0.70 | 0.71 |
| SciBERT | 0.68 | 0.69 | 0.81 | 0.88 | 0.76 | 0.78 | 0.89 | 0.89 | 0.75 | 0.76 |

Supplemental Table 1: Effect of target term delimiters on BioWiC^36^ test set accuracy. Left: models were trained using double quotes (‘‘…’’). Right: training using French quotes and spaces on both sides of the target terms (" «…» ").

The experiments explored two different approaches to preprocessing the sentences as input for the transformer-based models. In the first approach, the target terms within the corresponding contexts were enclosed in double quotes. In the second approach, to emphasize the target terms and separate them from the surrounding context, we enclose them within French quotation marks («...») and add spaces on either side. As shown in Supplemental Table 1, the models exhibited varying behaviors in predicting labels for different groups. However, the overall results showed only marginal differences between the models for the two preprocessing strategies, with both approaches achieving the same top performance of 76%. Further investigation of the effects of different sentence preprocessing strategies is planned as future work.
